# Endometriosis-associated hydrocele of the canal of Nuck with immunohistochemical confirmation: a case report

**DOI:** 10.1186/s13256-017-1522-x

**Published:** 2017-12-21

**Authors:** Kae Okoshi, Masaki Mizumoto, Koichi Kinoshita

**Affiliations:** Department of Surgery, The Japan Baptist Hospital, 47 Yamanomoto-cho, Kitashirakawa, Sakyo-ku, Kyoto, 606-8273 Japan

**Keywords:** Hydrocele of the canal of Nuck, Endometriosis, Podoplanin, Estrogen receptor

## Abstract

**Background:**

The canal of Nuck is an embryological vestige of the processus vaginalis, and presents a potential site for endometriosis seeding. Hydroceles in this region are a rare cause of inguinal swelling in females. In addition, endometriosis localized to the canal of Nuck is exceedingly rare.

**Case presentation:**

A 44-year-old Japanese woman presented with a painful mass overlying her right pubis. She underwent surgery to completely excise the mass. During surgery, division of the external oblique aponeurosis revealed a cyst that occupied the inguinal canal and it adhered to the transverse fascia, inguinal ligament, and pubic bone. The cyst was dissected from the round ligament, and the defect in the internal inguinal ring was repaired and reinforced with mesh. On macroscopic examination, the cyst had a heterogeneous fibrous aspect with dark brown inclusions. Microscopic examination revealed that the cyst was tortuous, lined by mesothelial-like cells, and accompanied by partial subcapsular hemorrhage. Endometrium-like tissue was observed in the cystic wall. Immunohistochemical staining for podoplanin confirmed the mesothelial origin of the cyst-lining cells. The epithelial cells and stromal cells were positive for estrogen receptors.

**Conclusions:**

In this case of an endometriosis-associated hydrocele of the canal of Nuck, the mesothelial origin of the cyst-lining cells and endometriosis were confirmed by positive immunohistochemical staining for podoplanin and estrogen receptors, respectively. We determined that hydrocele resection and reinforcement of the anterior inguinal canal wall (if necessary) are appropriate treatments for this condition.

## Background

A hydrocele of the canal of Nuck in females is analogous to the encysted hydrocele of the spermatic cord in men [1]. Inguinal hernia and inguinal endometriosis are possible causes of inguinal masses in females. As diagnoses of these masses by physical examination alone may be inconclusive, further evaluation using ultrasound [2–4] or magnetic resonance (MR) [3] imaging can help to establish definitive preoperative diagnoses and the extent of lesions. Complete surgical excision of the cyst is the standard treatment for this condition.

Endometriosis is a relatively common condition, although the estimated frequency varies by population. A previous study has reported that endometriosis affects 6 to 10% of reproductive-aged females, 50 to 60% of females (including teenage girls) with pelvic pain, and up to 50% of females with infertility [5]. Endometriosis may also occur in combination with inguinal hernia [6, 7] and hydrocele of the canal of Nuck [8, 9], but these cases are very rare. Here, we report a rare case of an endometriosis-associated hydrocele of the canal of Nuck. We also describe the use of immunohistochemical analysis to confirm the association of the hydrocele with endometriosis. This case report may help to increase awareness in general surgeons that inguinal masses in females may not only be due to hernia, but also due to endometriosis-associated hydroceles of the canal of Nuck.

## Case presentation

A 44-year-old premenopausal Japanese woman was referred to our department for a painful mass overlying her right pubis. She was a homemaker, and had first noticed the mass when she lifted several heavy books. She reported feeling the same symptom before, but did not remember when it had occurred. A review of her medical history showed that she had undergone laparoscopic surgery for intrapelvic endometriosis 10 years ago, but was not given any medication after being discharged. Her menstrual cycle had been irregular, but she denied fluctuation of the mass size in response to her menstrual cycle. She had a normal vaginal delivery 5 years before the present admission. She was a social drinker and a non-smoker of tobacco. Her family history revealed that her father had chronic renal failure and hypertension.

**Fig. 1:**
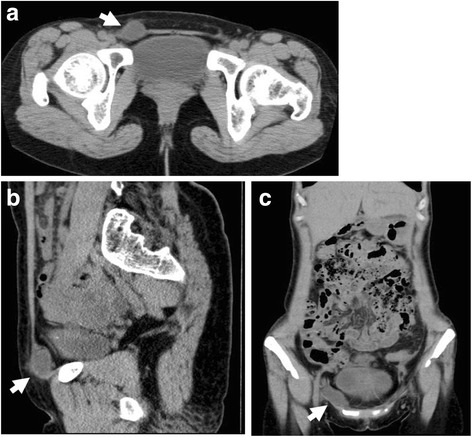
Computed tomography scans showing a round cystic lesion (*arrow*) within the right inguinal canal. **a** Transverse image. **b** Sagittal image. **c** Frontal image

A physical examination found a 3-cm subcutaneous thickening to the right of the midline over her right pubis, and the site was painful on palpation. A laboratory examination showed normal values in standard tests, except for a slightly elevated total bilirubin level of 1.4 mg/dL. Through computed tomography of her abdomen and pelvis, we identified a subcutaneous, well-defined cystic nodule measuring 2.7 × 2.2 cm in the inguinal canal (Fig. 1). There was no evidence of associated pelvic disease. This indicated that the lesion was either a hydrocele of the canal of Nuck, endometriosis, or an incarcerated ovary or omentum. She was admitted for surgery 18 days after the initial presentation. At admission, her blood pressure was 112/64 mmHg, pulse was 73/minute, and body temperature was 36.2 °C. A physical and neurological examination revealed no abnormalities except for the painful mass overlying her right pubis.

**Fig. 2:**
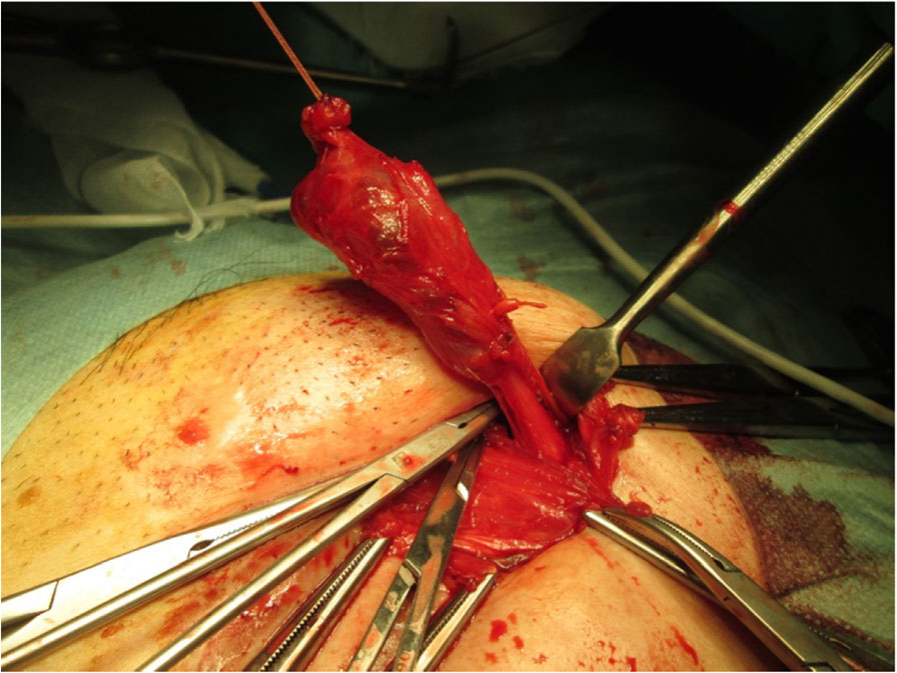
Intraoperative findings showing that the cyst, which contained dark brown fluid, strongly adhered to the round ligament of the uterus

The mass was completely excised through anterior open surgery. Division of the external oblique aponeurosis revealed a cyst filled with dark brown fluid that occupied the inguinal canal; the cyst adhered to the transverse fascia, inguinal ligament, and pubic bone. No hernia sac was detected. The cyst was dissected from the round ligament (Fig. 2), and the defect of the internal inguinal ring was repaired and reinforced with mesh. On macroscopic examination, the cyst measured approximately 5.0 × 3.0 × 2.0 cm, and had a heterogeneous fibrous aspect.

**Fig. 3:**
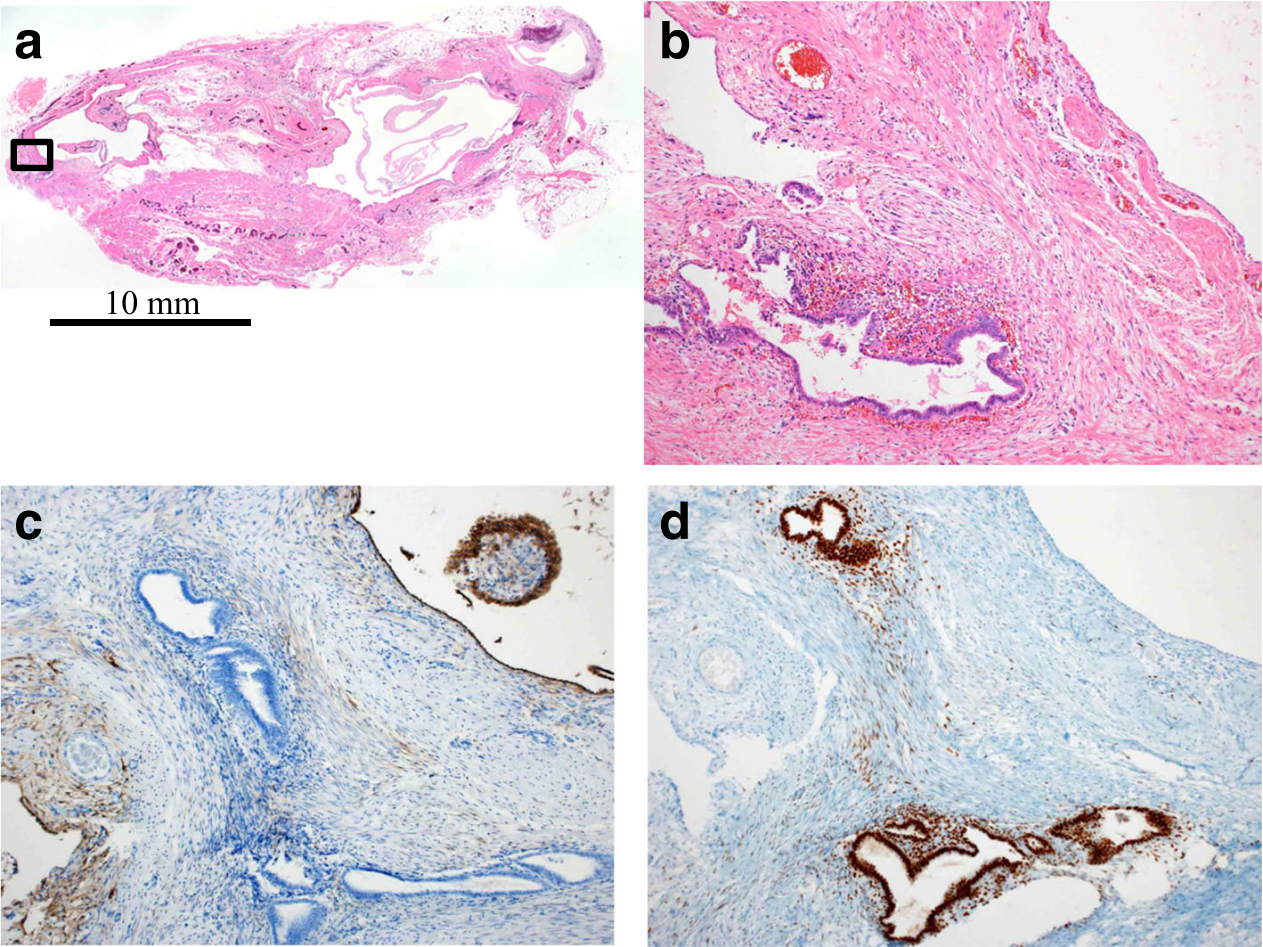
Histological examination of the excised cyst. **a** The cyst was tortuous (hematoxylin and eosin staining; low magnification). **b** Tissue is evident, but the cyst-lining cells are unclear; endometrium-like tissue is observed in the cystic wall (hematoxylin and eosin staining; high magnification). **c** Podoplanin-positive cyst-lining cells are clearly observed after immunohistochemical staining (immunoperoxidase stain with hematoxylin counterstain; high magnification). **d** Epithelial cells and stromal cells are positive for estrogen receptors after immunohistochemical staining (immunoperoxidase stain with hematoxylin counterstain; high magnification). **b**, **c**, and **d** are magnified images of the area within the frame in (**a**)

A histologic examination showed that the tortuous cyst was lined with mesothelial-like cells, and was accompanied by partial subcapsular hemorrhage (Fig. 3a, b). Endometrium-like tissue was observed in the cystic wall (Fig. 3b). No atypical cells were observed. Immunohistochemical analysis using immunoperoxidase stain with hematoxylin counterstain revealed that the cyst-lining cells were positive for podoplanin (D2-40) (Fig. 3c). In addition, the glandular epithelial cells and stromal cells were positive for estrogen receptors (Fig. 3d).

Her postoperative course was uneventful, and she was eventually discharged on the second postoperative day. She had not experienced any recurrent mass in the inguinal region 17 months after surgery.

## Discussion

This report describes a rare case of immunohistochemically confirmed endometriosis-associated hydrocele of the canal of Nuck in a 44-year-old Japanese woman. Endometriosis is a chronic estrogen-dependent condition that affects 6 to 10% of reproductive-aged females [5]. While ectopic endometrial tissue may appear anywhere in the body, implants tend to be located in the pelvic region. As a result, extrapelvic endometriosis is relatively rare. Scott and Te Linde [10] conducted an analysis of 516 endometriosis cases that included only four cases (0.8%) of inguinal endometriosis; among these, three cases involved the inguinal portion of the round ligament and one case involved the canal of Nuck. In addition, Jimenez and Miles [11] reported the incidence of endometriosis in the extraperitoneal portion of the round ligament to be 0.42%. Some researchers have described the diagnostic difficulty of inguinal endometriosis, and the most common preoperative diagnosis is inguinal hernia [11, 12].

To the best of our knowledge, few reports have conducted an immunohistochemical confirmation of endometriosis-associated hydroceles of the canal of Nuck. Usuki *et al*. [13] reported that the cyst-lining cells of such hydroceles were positive for calretinin and podoplanin, and that the subepithelial stromal cells were positive for CD10. In our patient, the cyst-lining cells were positive for podoplanin and the stromal cells were positive for estrogen receptors. These findings verified that the hydrocele was associated with endometriosis.

Previous studies have reported the identification of hydroceles of the canal of Nuck through ultrasound [2, 3] and MR imaging [3, 14]. Ozel *et al*. [3] reported that sonography showed a tubular cystic structure with internal septae in the inguinal canal of a female with inguinal swelling, and MR imaging found that the mass was hypointense in T1-weighted imaging and hyperintense in T2-weighted imaging.

Because our patient had previously undergone laparoscopic surgery for intrapelvic endometriosis, we were able to consider the possibility that the inguinal swelling was due to recurrent endometriosis. However, she did not notice that the inguinal swelling was related to her menstrual cycle, and had requested surgical excision for pain relief.

Treatments for a hydrocele of the canal of Nuck include surgical excision of the cyst with closure of the inguinal internal ring defect [9] and simultaneous inguinal hernia repair if required [15]. Surgical excision is effective as it is both therapeutic and diagnostic [16]. When possible, a laparoscopic approach may be considered because of the advantage of a shorter recovery period [9]. In addition, a laparoscopic approach enables an examination for any associated intra-abdominal endometriosis. However, further studies and longer follow-up periods are needed to verify the superiority and cost-effectiveness of the laparoscopic approach over anterior open procedures.

Patients with inguinal endometriosis often undergo initial surgery by a general surgeon under a diagnosis of hernia [17]. General surgeons therefore need to be more aware that inguinal masses in females may not only be due to hernia, but also due to endometriosis-associated hydroceles of the canal of Nuck.

## Conclusions

We observed a case with an unusual manifestation of extraperitoneal endometriosis in a hydrocele of the canal of Nuck. Although this is a rare condition, it should be included in the differential diagnosis of swelling in the inguinal region in females. Radical surgical excision with simultaneous inguinal hernia repair (if needed) represents the best option among current treatment choices because it allows for precise histological examination of the excised cyst and reduces the risk of relapse.

## Abbreviation

MR: Magnetic resonance
